# Evidence for direct dopaminergic connections between substantia nigra pars compacta and thalamus in young healthy humans

**DOI:** 10.3389/fncir.2024.1522421

**Published:** 2025-01-09

**Authors:** Giovanni Cirillo, Giuseppina Caiazzo, Federica Franza, Mario Cirillo, Michele Papa, Fabrizio Esposito

**Affiliations:** ^1^Division of Human Anatomy, Laboratory of Morphology of Neuronal Networks & Systems Biology, Department of Mental and Physical Health and Preventive Medicine, University of Campania “Luigi Vanvitelli”, Naples, Italy; ^2^Department of Advanced Medical and Surgical Sciences, Advanced MRI Research Center, University of Campania "Luigi Vanvitelli", Naples, Italy

**Keywords:** dopaminergic system, thalamus, DTI, HARDI, nigro-thalamic pathway

## Abstract

The substantia nigra pars compacta (SNc), one of the main dopaminergic nuclei of the brain, exerts a regulatory function on the basal ganglia circuitry via the nigro-striatal pathway but its possible dopaminergic innervation of the thalamus has been only investigated in non-human primates. The impossibility of tract-tracing studies in humans has boosted advanced MRI techniques and multi-shell high-angular resolution diffusion MRI (MS-HARDI) has promised to shed more light on the structural connectivity of subcortical structures. Here, we estimated the possible dopaminergic innervation of the human thalamus via an MS-HARDI tractography of the SNc in healthy human young adults. Two MRI data sets were serially acquired using MS-HARDI schemes from ADNI and HCP neuroimaging initiatives in a group of 10 healthy human subjects (5 males, age range: 25–30 years). High resolution 3D-T1 images were independently acquired to individually segment the thalamus and the SNc. Starting from whole-brain probabilistic tractography, all streamlines through the SNc reaching the thalamus were counted, separately for each hemisphere, after excluding streamlines through the substantia nigra pars reticulata and all those reaching the basal ganglia, the cerebellum and the cortex. We found a reproducible structural connectivity between the SNc and the thalamus, with an average of ~12% of the total number of streamlines encompassing the SNc and terminating in the thalamus, with no other major subcortical or cortical structures involved. The first principal component map of dopamine receptor density from a normative PET image data set suggested similar dopamine levels across SNc and thalamus. This is the first quantitative report from in-vivo measurements in humans supporting the presence of a direct nigro-thalamic dopaminergic projection. While histological validation and concurrent PET-MRI remains needed for ultimate proofing of existence, given the potential role of this pathway, the possibility to achieve a good reproducibility of these measurements in humans might enable the monitoring of dopaminergic-related disorders, towards targeted personalized therapies.

## 1 Introduction

Dopaminergic innervation of the brain is essential for motor control and higher cognitive functions including working memory, decision-making, reward-related learning, as well as emotion and motivational processing (Bayer and Glimcher, 2005; Lisman and Grace, 2005; Bromberg-Martin et al., 2010; Glimcher, 2011). According to the terminology of Dahlstroem and Fuxe (1964), dopaminergic nuclei of the brain include the group A9 (substantia nigra pars compacta, SNc), A10 (ventral tegmental area, VTA), A8 (retrorubral area) and A12 (arcuate nucleus of hypothalamus). Other minor dopaminergic centers are in the hypothalamus, precisely in the supraoptic/paraventricular region (A15), in the periventricular region (A14) and lateral hypothalamus (A13), in the periaqueductal gray (PAG) matter of the caudal hypothalamus, midbrain and pons (A11) and in lateral parabrachial nucleus (LPbn) at the ponto-mesencephalic junction (Sánchez-González et al., 2005). Altogether, these centers provide dopaminergic innervation of the striatum, the cerebral cortex, the nucleus accumbens and the amygdala (Zhang et al., 2017).

Substantia nigra (SN) is the largest cell mass of the midbrain, located between the tegmentum and the cerebral peduncle. The dorso-medial, cell-rich pars compacta (SNc) is dopaminergic whilst the ventro-lateral, less cellular, pars reticulata (SNr) is mainly GABAergic. In humans, SNc has been further divided into two parts, the ventral and the dorsal tiers, that are different in term of morphological features and connections. The dorsal tier cells continue medially with the adjacent VTA and mainly project to the limbic areas of the brain (ventral striatum, nucleus accumbens, cingulate and orbitofrontal cortex) whilst ventral tier cells mainly project to the dorsal striatum (caudate and putamen nuclei) (Quartarone et al., 2020; Carmichael et al., 2021; Bingham et al., 2023). However, the demonstration of a bidirectional cortico-nigral pathway in humans through MRI-based tractographic techniques has further widen the network of the SN (Cacciola et al., 2016). Evidence has demonstrated the connection of the SN with several brain structures such as primary sensory cortex, premotor cortex, temporal-occipital lobes, pontine basis and anterior lobe of cerebellum (Kwon and Jang, 2014). In rodents, primates and humans, dopaminergic endings have been found also in the thalamus by immunohistochemistry and retrograde tract-tracing techniques (Sánchez-González et al., 2005; Anaya-Martinez et al., 2006). Evidence in the rat brain has reported that nearly half of the SNc dopaminergic neurons project axons to the reticular nucleus of the thalamus and send branches to either the striatum or the globus pallidus. In *Macaca mulatta* and *Macaca nemestrina*, the most densely innervated thalamic regions are the limbic midline nuclei, the higher-order medio-dorsal (MD) and latero-posterior (LP) nuclei, and the motor ventro-lateral (VL) nucleus. These dopaminergic fibers originate bilaterally in multiple dopaminergic neuronal populations of the hypothalamus, PAG, ventral mesencephalon and LPbN. However, it has been never investigated the contribution of SNc fibers to dopaminergic innervation of the thalamus.

Based on these data, or lack thereof, we sought to expressly interrogate the connections of SNc with the thalamus by advanced MRI tractographic techniques using multi-shell (MS) high angular resolution diffusion imaging (HARDI) in humans.

More conventional diffusion tensor imaging (DTI) enables the analysis of anisotropic water motion in white matter and the non-invasive reconstruction and visualization of white matter fiber bundles (Mormina et al., 2015), estimating the connectivity patterns between distinct brain regions (Basser et al., 1994; Le Bihan and Johansen-Berg, 2012). Although suitable for large-scale whole-brain structural investigations exploring neural connectivity in normal subjects (Parker and Alexander, 2005; Jbabdi and Johansen-Berg, 2011; Jbabdi et al., 2015), DTI exhibits some limitations in exploring white matter (WM) regions with multiple fiber orientations and several associative fiber tracts (Behrens et al., 2007; Jones and Cercignani, 2010; Farquharson et al., 2013). Particularly, using DTI to separate fiber tracts through the SNc reaching the thalamus via the subthalamic area would be challenging, if not impractical, with current imaging resolution achievable with clinical MRI scanner (typically 2x2x2 mm), as demonstrated by a recent ex-vivo study (Oishi et al., 2020). However, in the last two decades, this limitation has also stimulated the adoption of higher order diffusion models, which are more sensitive to intravoxel orientation heterogeneity (Behrens et al., 2007; Caiazzo et al., 2016), especially if combined with data acquisition strategies that allow combining multiple shells to improve estimates of several fiber orientations within a voxel (Jbabdi et al., 2012).

To date, only few DTI studies have focused on the connectivity of SN in humans (Menke et al., 2010; Chowdhury et al., 2013; Kwon et al., 2021) suggesting that:

- SNc and SNr are both likely connected to cerebral cortex by means of the thalamus.
- SNr gives rise to the lateral and medial nigrothalamic tracts: the first proceeds along the surface of the thalamus through the internal capsule and reticular nucleus (Rt) to the superior part of the anterior thalamus, then penetrates toward the dorsal part of the ventral anterior (VA) and ventral lateral posterior internal part (VLpi) nuclei; the medial tract, originating from the caudal SNr, sends a tributary to the ventral medial nucleus (VM).
- SNc is highly connected with the prefrontal cortex.

Therefore, here we used for the first time MS data sets and an HARDI-based probabilistic tractography to investigate the possibility that a non-negligible amount of fiber connections through the SNc would directly reach, not only the basal ganglia, but also the thalamus, thereby suggesting the possibility of an additional, parallel dopaminergic circuit in the human brain.

While it is well known that tractography results may still suffer from possible false positives, and because there are currently no unique prescriptions for acquisition parameters ensuring an optimal control of false positives, here we independently and consequently acquired two different data sets in ten healthy young human subjects, using parameters from two different worldwide initiatives (ADNI, HCP). These parameters have been previously shared to the neuroimaging community to minimize the inter-scanner variance in multi-center studies, thereby increasing the average quality of big data bases. In our work, we intended to vary some of the major DWI sequence parameters (i.e., the diffusion weighting and the number of gradient directions per each shell) before applying the same tractography pipeline, in such a way to collect repeated measures of the same connectivity estimates, in each subject, and possibly increase the reliability and power of our findings despite the small sample size. While it is not yet possible to confirm with tractography results only the existence of fiber bundles that have not been histologically proven, in the present work, we also provide the first stable tractography results which would be compatible with the role that the thalamus is thought to play in parkinsonian and essential tremor patients (Milosevic et al., 2018), possibly reviving the interest for future more advanced histological and ex-vivo studies expressly targeted to confirm the existence of these connections.

## 2 Materials and methods

### 2.1 Subjects

Right-handed healthy young adult subjects were enrolled by word of mouth. Ten neurologically and cognitively normal subjects (5 M, 5F; mean age ± st. dev. 23.8 ± 3.65 years) were included in the study. This work was carried out in accordance with The Code of Ethics of the World Medical Association (Declaration of Helsinki) for experiments involving humans. Ethics approval was obtained from the Ethics Committee of the University of Campania “Luigi Vanvitelli.” Written consent was obtained from each participant.

### 2.2 MRI image acquisition

MRI was performed on a 3 Tesla scanner (Discovery MR750, General Electric, United States) equipped with a 32-channel receive-only head–neck coil. The imaging protocol included the following series:

1. 3D T1-weighted inversion recovery fast spoiled gradient recalled echo (3D-IR-FSPGR) with sagittal reconstruction (TR = 6,912 ms, IT = 650 ms, TE = 2.996 ms, flip angle = 9°, voxel size = 1 × 1 × 1 mm) for high resolution anatomical reference.
2. Multi-shell Diffusion MRI (scheme MS1): 2D diffusion weighted spin-echo echo-planar imaging series (94 slices, TR = 5,5 s, TE = 0.075 s, isotropic voxel size 2.0 mm, 30 gradient directions with b value = 700 s/mm2, 30 gradient directions with b value = 1,000 s/mm2, 64 gradient directions with b value = 2000 s/mm^2^) and an additional series with opposite polarity of the phase encoding direction were acquired for distortion correction.
3. Multi-shell Diffusion MRI (scheme MS2): 2D diffusion weighted spin-echo echo-planar imaging series (80 slices, TR = 5,5 s, TE = 0.082 s, isotropic voxel size 2.0 mm, 30 gradient directions with b value = 1,000 s/mm^2^, 60 gradient directions with b value = 2000 s/mm^2^, 90 gradient directions with b value = 3,000 s/mm^2^) and an additional series with opposite polarity of the phase encoding direction were acquired for distortion correction.

### 2.3 MRI tractography

Masks of SNc for the right (R) and left (L) hemisphere were extracted from the SN probabilistic atlas (Safai et al., 2020). Masks for the left (L) and right (R) substantia nigra pars reticulata (SNr), thalamus (Th), caudate (Cd), putamen (Pt), globus pallidum (Gp), cerebellum (Cr) and vermis (Vr) were extracted from the AAL3 atlas (Rolls et al., 2020). Masks for the left and right dentate nucleus (Dn) were extracted from the cerebellar probabilistic atlas (Diedrichsen et al., 2009) and a mask for the whole cortex (Ct) was extracted from the probabilistic Harvard-Oxford cortical FSL atlas.^1^ All masks were initially obtained from the atlases in the standard MNI space (see Supplementary Figures S1, S2) and then non-linearly back-transformed to native diffusion space using 3D-T1 images from each individual subject and the FLIRT and FNIRT tools of FSL (see text footnote 1). All the individual SNc and SNr masks were visually checked in the subject native space (see, e.g., Supplementary Figure S3).

Multi-shell diffusion-weighted MRI data sets (MS1, MS2) were processed using tools from FSL (see text footnote 1) and MRtrix3 toolbox.^2^ MS1 and MS2 series were preliminary denoised and corrected for possible artifacts. More specifically, the denoise tool of MRtrix3 was used to improve signal-to-noise ratio by removing noise-only principal components according to the random matrix theory (Veraart et al., 2016; Cordero-Grande et al., 2019). Brain extraction, echo-planar image unwarping, eddy current distortion and motion correction were performed using the FSL tools bet and eddy (Andersson and Sotiropoulos, 2016). Particularly, the eddy-quad tool was used to generate a quality control report to verify that the absolute motion parameters (mm) were lower than the image voxel dimension (2 mm) and that the SNR of all diffusion images were above 15 (Dell’Acqua and Tournier, 2019), for all subjects.

Multi-shell multi-tissue (MSMT) constrained spherical deconvolution (CSD) was applied to MS1 and MS2 data sets in MRtrix3 to obtain the fiber orientation density (FOD) function at each voxel (Jeurissen et al., 2014). For each data set, the obtained FOD ranges were normalized across subjects using the “mtnormalise” tool of MRItrix3 (Dhollander et al., 2021). Then, for each subject and data set, the second-order integration over fiber orientation distribution (iFOD2) algorithm (Tournier et al., 2010) within the anatomically-constrained tractography (ACT) framework (Smith et al., 2012) was used to generate a 10 million streamlines whole brain probabilistic tractography with dynamic determination of seed points (MRItrix command: *tckgen*). The ACT option ensured that all traced streamlines terminating in cerebro-spinal fluid (CSF) were dismissed. To this purpose, we performed tissue segmentation on 3D T1-weighted images using the *5ttgen* command of MRtrix3 tool. As further options: backtracking was enabled (thereby tracks are eventually truncated and re-tracked if a poor structural termination is encountered), FOD amplitude threshold was set to 0.06 and maximum length for streamlines was set to 250 mm. Spherical-deconvolution informed filtering of tractograms (SIFT) was finally applied to reduce the number of streamlines to 1 million, thereby filtering out most anatomically implausible streamlines (Smith et al., 2013). At this stage, no inclusion or exclusion criteria was applied, i.e., the *-include spec* option was not used in the MRtrix command *tckgen*. It is only in the later step, i.e., when the reconstruction of all streamlines is completed, that the ROI selection criteria are applied, resulting in the mere filtering of the whole-brain tractograms. To this purpose, we used the *-include spec* and *ends_only* options of the MRtrix command *tckedit.* In this way, starting from previously reconstructed streamlines, we first selected only streamlines encompassing the SNc (originating from, and terminating within the imaging slab) and then, we selected the streamlines terminating in the thalamus (but not in the other regions).

To extract an MRI-derived tractography metric, first the number of streamlines between the SNc and the thalamus for both hemispheres was separately counted (and compared, see section 2.4). Then, these counts were percent-normalized by the number of whole-brain streamlines encompassing the SNc in such a way to obtain an individual percent-normalized count of SNc-Th connections per subject and data set among the whole set of SNc brain connections. Here, only the SNc connections directly reaching, and terminating within, the thalamus, were considered, by specifying the Th masks as region of termination and all other masks as regions of exclusion. For each subject and data set, the number of SNc-Th streamlines was percent-normalized to the global number of SNc streamlines from the whole-brain tractography (Plantinga et al., 2016). To also report the individual reproducibility of the metric between the two schemes, the inter-subject correlation of the percent-normalized connectivity was calculated. Finally, population-level density tracts on the MNI space were generated as t-statistic maps in MNI space (i) using the “tckmap” command of MRtrix with the “-contrast tdi” option to obtain the individual track density maps in the native space and (ii) registering each map to the MNI space to capture the mean track density and the inter-individual variability in the reconstructions at each voxel. Normalization parameters as obtained for the individual estimated MNI transformations were applied to individual track density maps for group overlap. The resulting t-statistic maps were thresholded between 2 and 10 and overlaid to the MNI template, not to suggest the possible existence of this pathway at each voxel but only to describe the trend in the average density of tracks at a voxel level normalized to the standard error. Therefore, a t-value above 2 would not ensure about the existence of the pathway through each single voxel but only depicts where the average density is at least twice the standard error of the same descriptor in our sample.

### 2.4 Statistical analysis

The statistical analysis of MRI-derived measures was performed in in MATLAB R2023a (Mathworks, Inc., http://www.mathworks.com) using functions from the statistics toolbox. First, counts were expressed by mean ± standard deviation and compared between measurements and hemispheres. Two-tailed one-sample (paired) t-tests were performed to compare absolute counts and tractometric estimates between MS-HARDI measurements and between hemispheres (within subject). Particularly, as we obtained separate estimates for each hemisphere, we also investigated the laterality by comparing the estimates from different hemispheres with paired t-tests. Given that we derived the same SNc-Th connectivity metrics from two different MS-HARDI protocols, we finally assessed the differences in these MRI estimates between acquisition protocols using paired t-tests. Instead, for each MS-HARDI protocol, one-sample signed-rank tests were used to test whether the median percentage of selected SNc-Th streamlines was significantly above 0, 5 and 10%. Here we compared median values of percent-normalized estimates to a set of thresholds because the median is a centrality descriptor more robust to anomalous values and better reflects the central tendency of the data without prior assumptions on the distribution of inter-subject variability, given also the small size of the analyzed cohort.

### 2.5 Normative PET image analysis

Because tractography-derived streamlines are not labelled to any specific neurotransmitter, a descriptive image of normative regional variations in dopamine receptor densities was obtained using an open-access PET dataset which has been recently described in the literature (Hansen et al., 2022). More specifically, we downloaded PET images acquired in healthy participants which were already averaged for dopamine receptor (D1, D2), transporter (DAT) and for F-DOPA scans. These images were all registered to the MNI template, thereby the first principal component of receptor density was calculated to obtain a regional quantification of dopamine receptor similarities across the whole brain, including SNc and Thalamus. The resulting z-score map was back-transformed to the native MRI space of one individual representative subject to descriptively compare tractograms and dopamine receptor density distributions on the same brain.

## 3 Results

**Figure 1:**
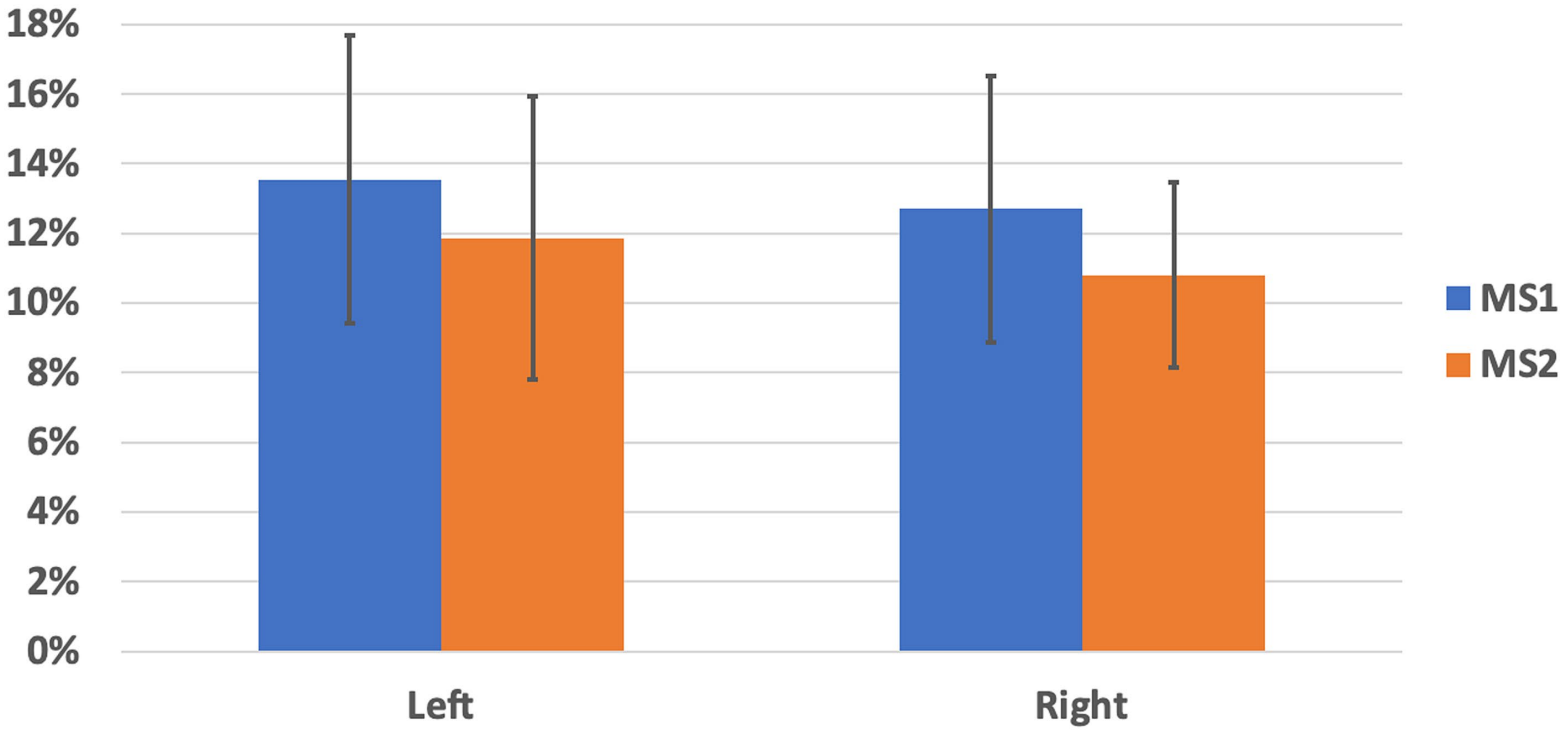
Percentage of streamlines directly connecting SNc and thalamus, normalized to the total count of whole-brain streamlines encompassing the SNc in each hemisphere. Mean and standard deviation are shown for left and right hemisphere and for the two MS-HARDI data sets (MS1, MS2).

First, we report the streamline counts for both measurements and hemispheres: whole-brain probabilistic tractography applied to MS1 data sets allowed tracing a total of 1349.30 ± 138.30 (left hemisphere) and 1143.20 ± 183.47 (right hemisphere) through the SNc masks. Whole-brain probabilistic tractography applied to MS2 data sets allowed tracing a total of 1341.30 ± 146.12 (left hemisphere) and 1257.90 ± 228.16 (right hemisphere) through the SNc masks. For both hemispheres, these counts did not significantly differ between the two schemes (MS1 vs. MS2, paired t-test, left: *p* = 0.90, right: *p* = 0.23), albeit the total count of SNc streamlines was significantly higher for the left hemisphere, compared to the right, for the MS1 scheme (left vs. right, paired t-test, *p* = 0.0034), but not for the MS2 scheme (left vs. right, paired t-test, *p* = 0.27). Therefore, for both MS1 and MS2 data sets, the percentage of SNc-Th streamlines depicting a direct anatomical connectivity pathway between SNc and Th regions was taken as an index of connectivity. For MS1 data sets, this index amounted to 13.55 ± 4.13% (left hemisphere) and 12.70 ± 3.81% (right hemisphere). For MS2 data sets, the percentage of direct SNc-Th streamlines was 11.87 ± 4.05% (left hemisphere) and 10.81% ± 2.66% (right hemisphere). These results are summarized in Figure 1. For both hemispheres and MS data sets, the median percentage of direct SNc-Th streamlines was significantly above 0% (one-sided signed rank test: *p* < 0.001) and significantly above 5% (one-sided signed rank test: *p* < 0.05). For both hemispheres, the median percentage of direct SNc-Th streamlines estimated from MS1 data set was also significantly above 10% (one-sided signed rank test: left *p* = 0.0195, right: *p* = 0.0137). For both hemispheres, these estimates were tendentially higher for MS1, compared to MS2, data sets, albeit the statistical significance of these differences was only close to the conventional threshold of *p* = 0.05 (MS1 vs. MS2, paired t-test, left: *p* = 0.062, right: *p* = 0.053). As for the reproducibility between MS1 and MS2 schemes, the inter-subject correlation of the connectivity indices was 0.78 for the left hemisphere and 0.50 for the right hemisphere. However, for both data sets, the percentage of direct SNc-Th streamlines was not significantly different between left and right hemispheres (paired t-test, MS1: *p* = 0.29, M2: *p* = 024).

**Figure 2:**
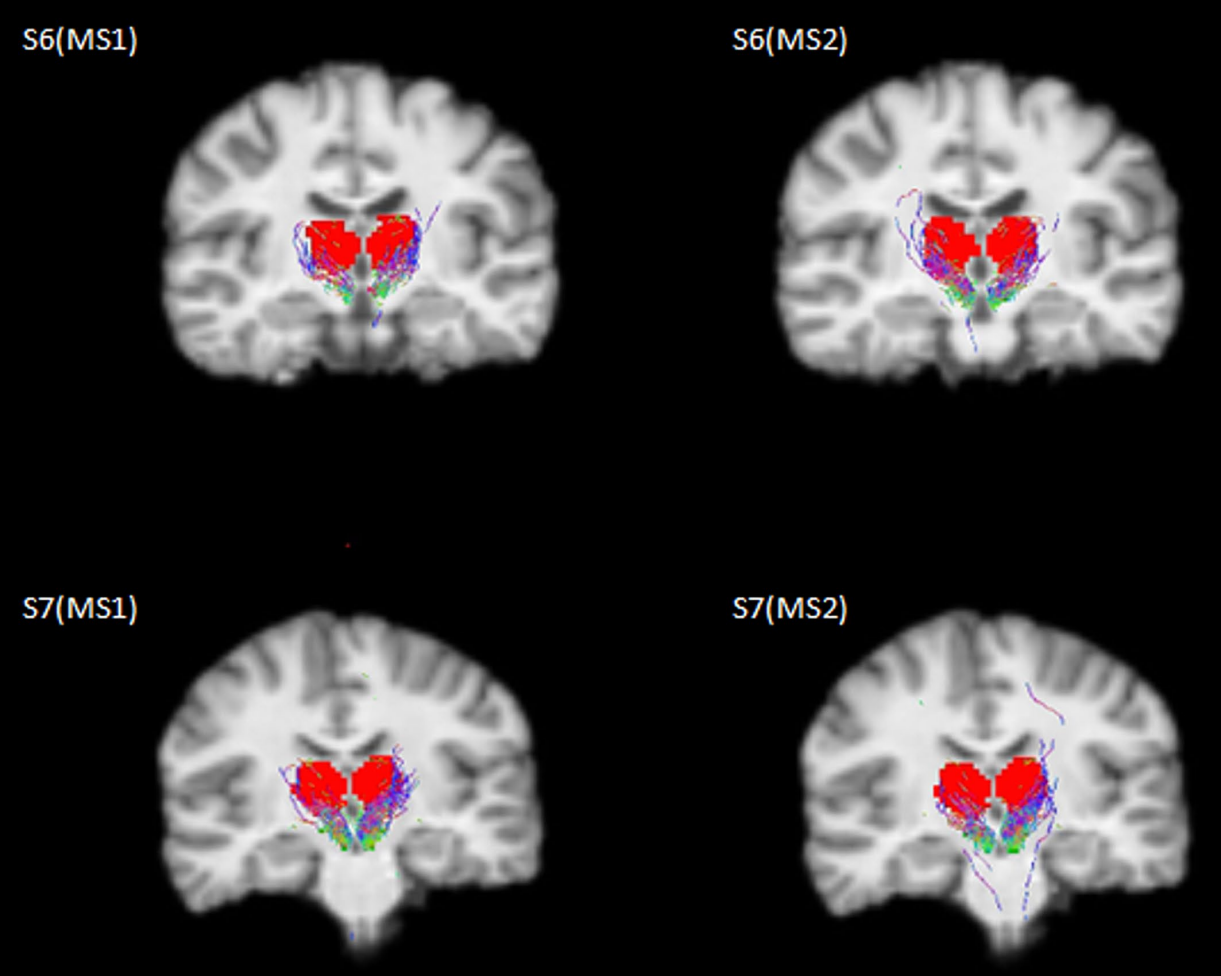
Selected streamlines directly connecting SNc (green mask) and thalamus (red mask) overlaid on a coronal slice from the 3D T1 scan for two representative subjects (S6: male, 21y; S7: female, 22y) as obtained from the whole-brain probabilistic tractography of two MS-HARDI data sets (MS1, MS2) in the native diffusion space.

**Figure 3:**
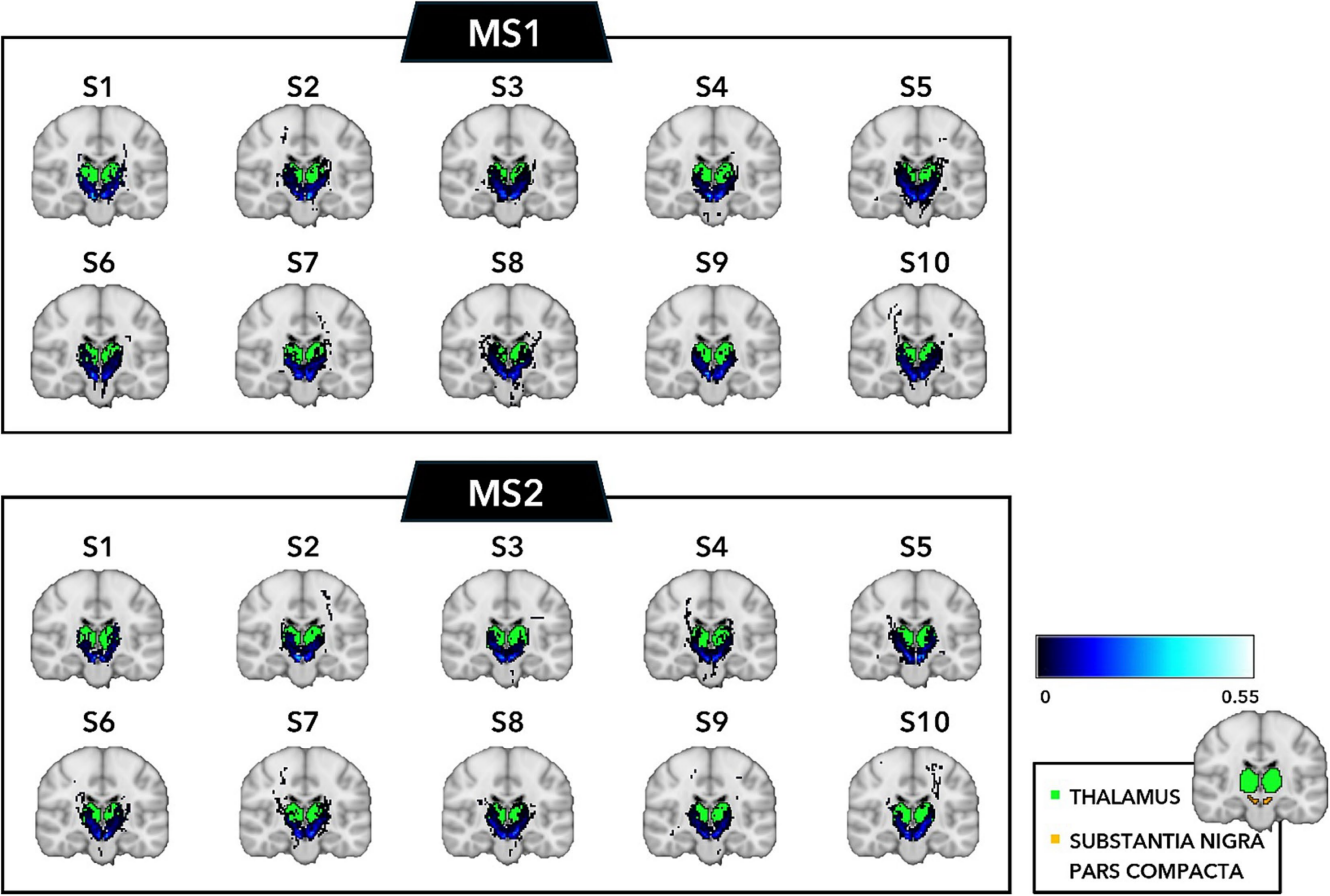
Individual track density maps in the MNI space for the direct connection between SNc and thalamus overlaid on the same coronal slice of the MNI T1 template as obtained from the whole-brain tractography of all individual subjects for the two MS-DWI data sets (MS1, MS2) and the subsequent registration to the MNI template.

**Figure 4:**
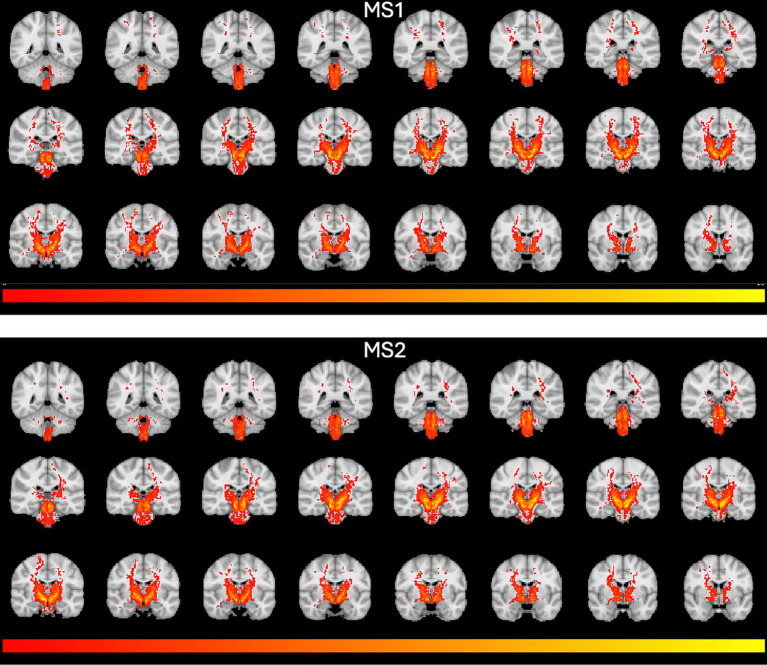
Population-level tractography maps. T-statistics group maps were obtained from individual track density maps in Figure 1 and overlaid on the MNI T1 template. T-maps were thresholded between t = 2 and t = 10.

The selected SNc streamlines contributing to the direct SNc-Th connectivity in both hemispheres, as obtained from both MS1 and MS2 series, are displayed for two representative individual subjects (a male and a female) in Figure 2 (native space) and for all ten subjects in Figure 3 (in MNI space). A more complete description of the anatomical course of the bundle of interest is also displayed in the native space of one representative subject (MS1 data set) as several slices across different planes (axial, coronal, sagittal) using a light box visualization (see Supplementary Figures S4–S6). Population-level reconstructions of the direct SNc-Th tracts (as t-statistic maps) are displayed in Figure 4.

Finally, this connectivity is also displayed for the left hemisphere using MS1 data from one representative subject after overlaying the first principal component map of receptor density expressing (in spatial z-scores) the regional variations in receptor density images as obtained from the PET normative database for four different tracers (D1, D2, DAT, FDOPA). Scaling this map between zero and five was sufficient to highlight similar levels in receptor densities across SNc and thalamus (1 ≤ z ≤ 5), such levels being intermediate between lower values (z < 1) across the cerebral cortex and higher values (z > 5) within the basal ganglia (Supplementary Figure S7).

## 4 Discussion

The widespread dopaminergic innervation of the thalamus has represented a novelty in the study of the brain’s hodology (Craven, 2005) and has been even related to an evolutionary complexity of the higher brain centers (i.e., cerebral cortex, basal ganglia, amygdala and accumbens), supporting higher brain functions (García-Cabezas et al., 2007; Hwang et al., 2017; Shine, 2021).

Retrograde tract tracing studies in the macaque brain have shown that dopaminergic innervation of the thalamus originates bilaterally in hypothalamus, periaqueductal grey and ventral mesencephalon, thereby being involved in the modulation of wakefulness of the animal and current attentional and behavioral demands (Sánchez-González et al., 2005). The most densely dopamine-innervated thalamic nuclei are the midline (intralaminar), mediodorsal (MD), and latero posterior (LP) nuclei, specifically connected with associative fronto-parietal cortical regions. To date, the contribution of SNc to the dopaminergic innervation of the thalamus has not been investigated so far and was therefore the main purpose of this study.

Most of the efferent connections of the SNc are directed to the dorsal striatum (nigro-striatal pathway) or to the ventral striatum (meso-limbic pathway), according to the DeLong model of basal ganglia morpho-functional organization (Wichmann and DeLong, 2001). Briefly, dopamine modulates GABAergic medium spiny neurons (MSN) of the striatum (caudate and putamen), whose output is directed to the thalamus through two different systems (direct and indirect systems). The thalamus is also modulated by two hyperdirect pathways, based on a fast cortical modulation of the globus pallidus (GP) (Milardi et al., 2015) and subthalamic nucleus (STN) (Bingham et al., 2023). Recently, probabilistic CSD tractography on MRI allowed the confirmation of an extensive neural pathway running between the SN and cerebral cortex (mainly prefrontal areas) (Cacciola et al., 2016). In the current model, however, despite long-projecting nigro-cortical axons, thalamic nuclei do not appear as directly and specifically connected to the SNc.

**Figure 5:**
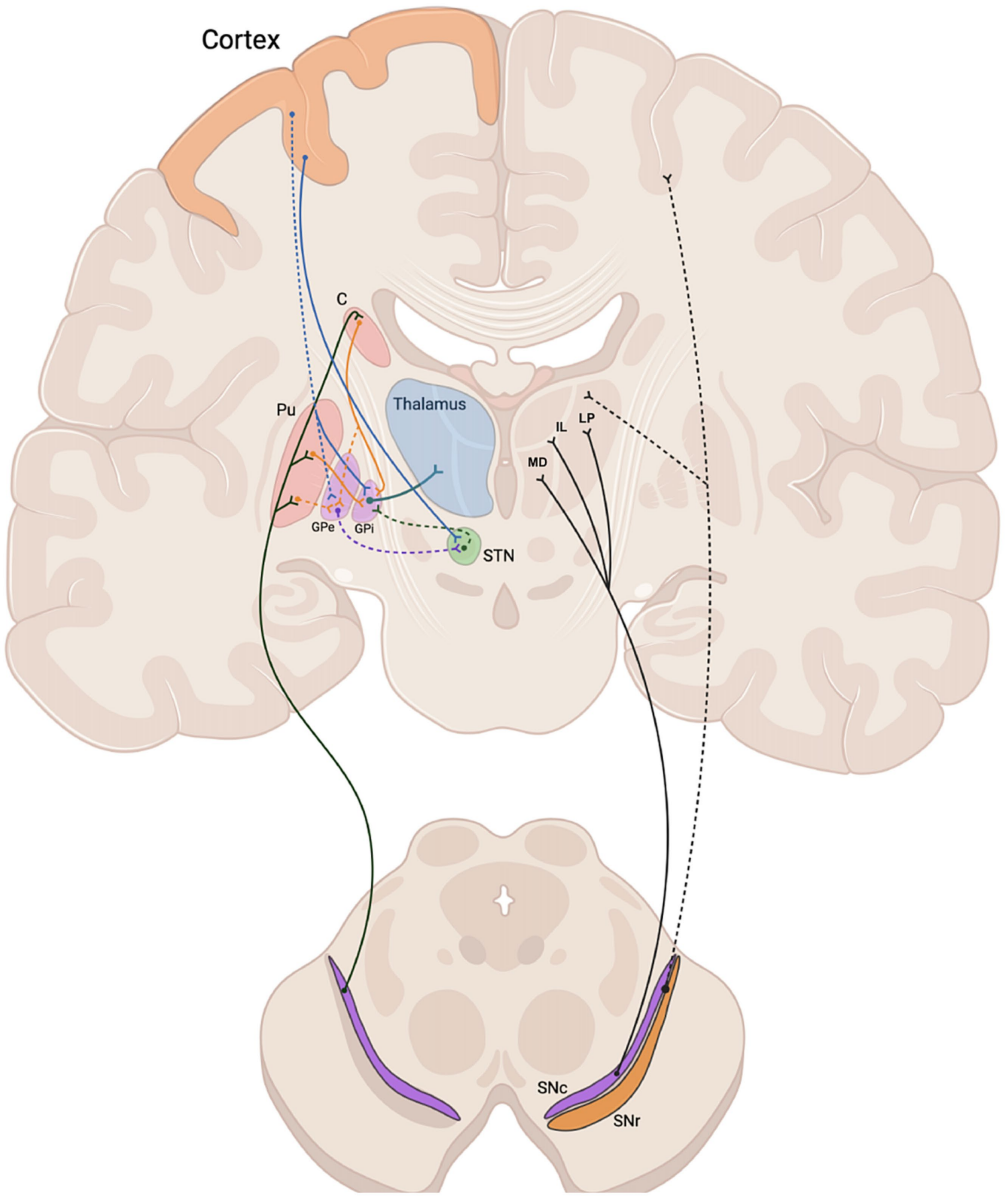
Schematic representation of the nigro-thalamic system. Efferent connections of the SNc to the dorsal striatum (caudate and putamen) (left) and to thalamic nuclei (MD, IL, LP) (right), together with nigro-cortical fibers (dashed black line). Direct, indirect and hyperdirect pathways are represented on the left side. C, caudate; Pu, putamen; MD, medio dorsal nucleus; IL, intralaminar nuclei; LP, latero posterior nucleus; GPe, external globus pallidus; GPi, internal globus pallidus; STN, subthalamic nucleus; SNc, substantia nigra pars compacta; SNr, substantia nigra pars reticulata.

Our results challenged this traditional model, demonstrating a relevant (~12%) and significant non-zero percentage of tracts from whole-brain probabilistic tractography of the SNc ending up in the thalamus in both cerebral hemispheres, after excluding SNr, caudate, putamen, globus pallidum, cerebellum, vermis, dentate nuclei and the entire cerebral cortex as possible terminations. Reporting the percentage of SNc streamlines is typical for this kind of studies. For example, referring to similar studies aiming at reporting quantitative estimates of dopaminergic pathways from high angula r resolution dMRI data, Plantinga and colleagues also assessed (and reported) the percentages of total track counts through SNc for the targeted structures (Plantinga et al., 2016). Here we (repeatedly) scanned 10 brains and therefore also considered the average percentage of total track counts through SNc after selecting the tracks terminating in the target structure (thalamus). However, as the average is only a statistical descriptor, and the number of brains is relatively low, we also statistically assess this finding after considering the inter-subject variability across the 10 brains and one-signed rank tests were performed to test whether the median estimates of these percentages were significantly above 0% and above 5%, obtaining that this result is not much likely to be due to false positives (*p* < 0.05) and therefore more likely to suggest (albeit not necessarily imply) the existence of this tract. Anyway, this finding, which was here replicated using two different MS-HARDI sequences on a clinical 3 T MRI scanner in the same cohort of ten adult human subjects, represents the first description of a pathway compatible with a direct anatomical connection between SNc and the thalamus, bypassing the striatum. Given the known nature of SNc neurons, this may therefore represent an additional route for dopaminergic regulation of the subcortical circuitry in humans (Figure 5).

Interestingly, when overlaying the first principal component map of dopamine receptor densities, as obtained from a normative database of PET images recently made available (Hansen et al., 2022), similar z-score levels were observed across SNc and thalamus masks used for tractography, above the cerebral cortex and below the basal ganglia. However, while the similarity in receptor types and levels across pairs of (sub)cortical regions seems to underly a shared axis of spatial variability between the intrinsic chemoarchitecture and functional or structural connectivity of the brain (Hänisch et al., 2023), this observation can only be taken as circumstantial evidence to, i.e., compatible with, but certainly not corroborating, the presented MRI findings.

The SNc-Th pathway identified by our analysis also appeared slightly leftwardly asymmetric, especially from the data acquired according to the MS1 scheme (albeit a trend was visible in both data sets). However, the individual reproducibility of the connectivity metric was only good for the left hemisphere (correlation higher than 0.7) and could only be considered fair for the right hemisphere (correlation between 0.4 and 0.7). In other studies (Parker et al., 2005; Barrick et al., 2006), brain asymmetry has been also highlighted and discussed as a matter of enduring scientific interest, some experts currently believing that both genetic and environmental factors may contribute to its development (Jahanshad et al., 2010).

The relevance of these data comes from clinical and experimental research focusing on thalamic abnormalities related to dopamine and its receptors in patients affected by schizophrenia (Wagner et al., 2015; Plavén-Sigray et al., 2022) and Parkinson’s disease (PD) (Rüb et al., 2002; Monje et al., 2020). The thalamus plays a central role in modulating cortical activity, and dopaminergic input may enhance its influence on cognitive processes such as attention, memory, and executive function. Notably, Mukherjee et al. (2021) provide compelling evidence for the functional relevance of dopaminergic modulation in the MD thalamus. This study identified a subpopulation of thalamic neurons expressing D2 receptors, which modulate prefrontal cortex (PFC) states through a non-linear gain control mechanism. Such modulation is critical for cognitive flexibility and behavioral adaptation, allowing dynamic adjustments to changing environmental demands. The structural findings presented here may provide the anatomical basis for such mechanisms. The potential pathways linking SNc to the thalamus could serve as substrates for dopaminergic regulation of thalamo-cortical circuits, influencing neural dynamics underlying cognitive flexibility. This aligns with broader theories of dopaminergic involvement in state-dependent cortical processing, as discussed in recent reviews (Scott et al., 2024; Wolff and Halassa, 2024), in which the thalamus’s role in facilitating adaptive behaviors by regulating cortical network states via dopaminergic signals has been discussed. Furthermore, the dopaminergic innervation of the thalamus could support mechanisms that integrate external sensory information with internal cognitive states, crucial for goal-directed behavior. By linking these structural findings to existing literature on thalamo-cortical interactions and dopaminergic modulation, this study not only advances our understanding of SNc-thalamus connectivity but also suggests its broader functional implications for cognitive and behavioral adaptability. Future studies exploring these connections *in vivo* will be crucial to validating these hypotheses and further elucidating the role of dopaminergic pathways in thalamo-cortical function.

Our MS-HARDI study regarding SNc connectivity has some limitations worth noting. First, we are aware that tractography does not provide a direct visualization of axons, allowing only a probabilistic representation of the most likely trajectories based on local water molecule diffusion, and does not establish the direction of the signal transmission. Even the connection index provided here enables an anatomically constrained quantitative analysis of the “relative amount” of structural connectivity existing between two structures, i.e., SNc and thalamus, after excluding several other structures, but cannot be associated with the likelihood of these tracts being existent. Particularly, given the resolution of the acquired images, possible distinct fiber tracts through the subthalamic area violating the anatomical constrains, i.e., not originating in SNc or not terminating in the thalamus, would be (wrongly) captured if identically aligned with fiber tracts originating in SNc, thereby contributing to false connections. However, to our latest knowledge, no in-vivo (human or primate) or ex-vivo DWI study used a similar high-order tractography to expressly interrogate (and possibly reconstruct) hypothetical tracts from the SNc to the thalamus (whether partially through the STN or not) with MS-HARDI data. In our study, despite the low (2 mm) resolution of the images, we had two repeated acquisitions in the same subjects and more than hundred directions sampled in total across three different shells and anyway, in the next future, we aim to couple this approach with post-mortem microsurgery dissection and/or tracer injection to confirm the existence of the hypothesized dopaminergic nigro-thalamic pathway. Second, tractography results might still suffer from reconstruction biases (i.e., possible false positive streamlines). Thereby, further investigations, using higher resolution acquisition and 7 T MRI scanners, are ongoing to validate these data and to identify, in combination with PET data, which specific thalamic nuclei are more targeted by such hypothesized direct SNc pathways. Third, it will be also crucially important to understand the possible clinical correlate of SNc-Th pathway, if any, disentangling the role of dopaminergic innervation of the thalamus in a proof-of-concept group of PD patients. Finally, our results, based on a relatively small sample of adult healthy subjects, need to be validated in a larger sample of healthy subjects to provide indications about normative populations, although results were here obtained individually on each single subject data sets and exhibited limited inter-subject variability and good overall consistency of the main finding with normative PET images of dopamine receptor density. It is also for this reason that all ROIs were retrieved from publicly available atlases. Particularly, even if manual segmentation remains the gold standard, SNc masks were initially generated using a special SNc probabilistic atlas which was originally generated from manually segmented 3D neuromelanin sensitive MRI images in 27 healthy control subjects and carefully validated on independent cohorts of healthy subjects (Safai et al., 2020).

In conclusion, despite the limitations, the illustrated tractographic approach would remain the only available technique to investigate structural neural connectivity between the SNc and the thalamus in-vivo and non-invasively in humans which would in principle also work on several clinical MRI scanners. The replication with two different MS-HARDI sequences derived from worldwide neuroimaging prescriptions demonstrated the compatibility of these findings with the existence of direct anatomical nigro-thalamic connections from SNc, allowing us to not discard in advance a potentially important piece of the puzzle toward a more extensive comprehension of the subcortical regulation and dopaminergic reward circuits beyond the basal ganglia, and paving the way for further morpho-functional investigations in healthy subjects and patients with PD or similar disorders.

## Data Availability

The raw data supporting the conclusions of this article will be made available by the authors, without undue reservation.

## Footnotes

^1^ https://www.fmrib.ox.ac.uk/fsl

^2^ https://www.mrtrix.org/
