# Supplementary material for: Evidence for direct dopaminergic connections between substantia nigra pars compacta and thalamus in young healthy humans
Source: Front Neural Circuits. 2025 Jan 9;18:1522421. doi: 10.3389/fncir.2024.1522421 (PMC11754968; doi:10.3389/fncir.2024.1522421)
Supplement: Supplementary file 1 [file Data_Sheet_1.docx]

Supplementary Material

## Supplementary Figures

**Supplementary figure 1**.


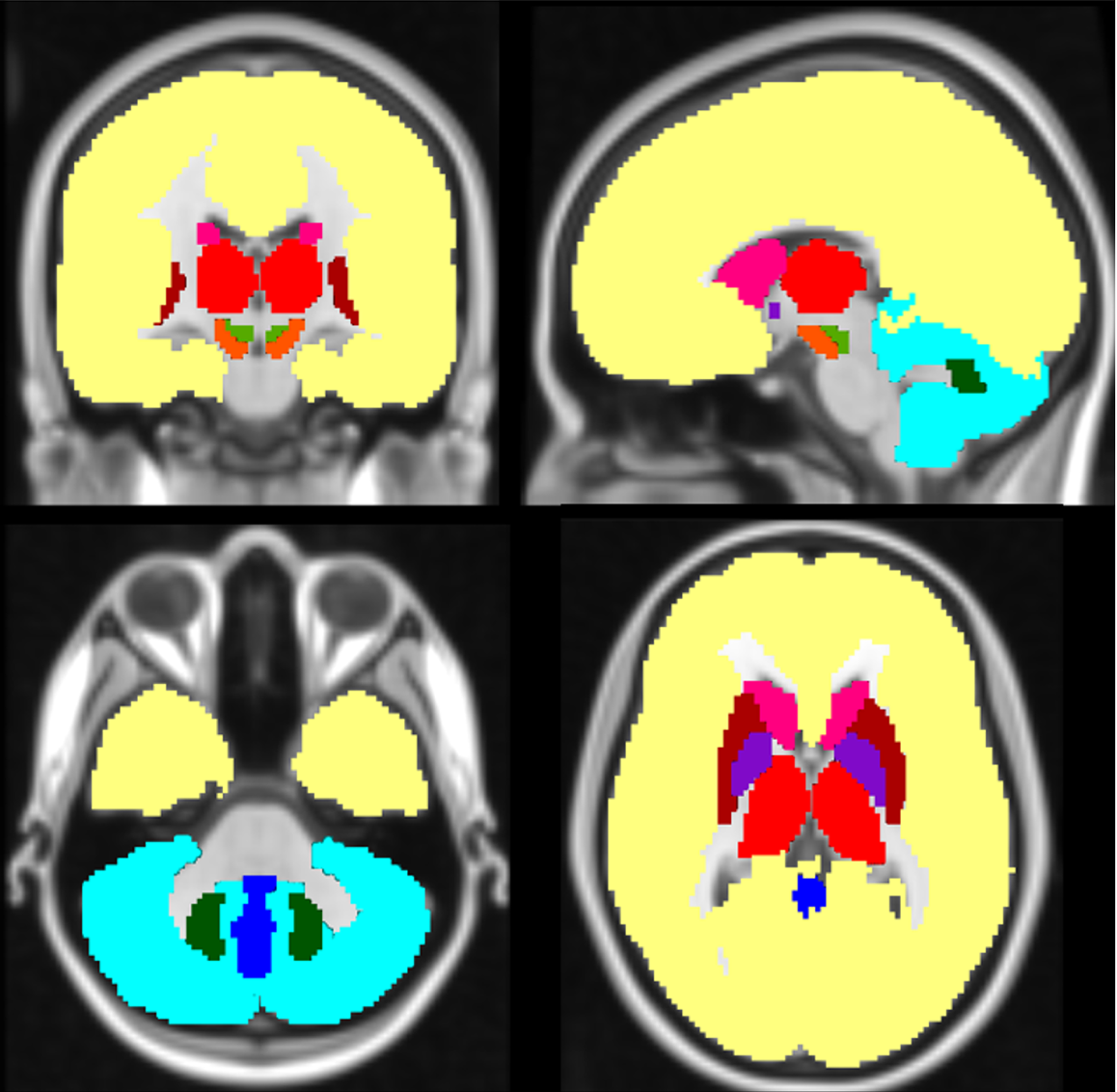


**Supplementary Figure 1.** Masks for the left (L) and right (R) substantia nigra pars compacta (orange) and reticulata (green), thalamus (red), caudate (purple), putamen (dark red), globus pallidum (purple), cerebellum (cian) and vermis (dark green) from the AAL3 atlas (Rolls et al., 2020) and mask for the whole cortex (yellow) from the probabilistic Harvard-Oxford cortical FSL atlas (<https://www.fmrib.ox.ac.uk/fsl>), in the standard MNI space.

**Supplementary figure 2**.


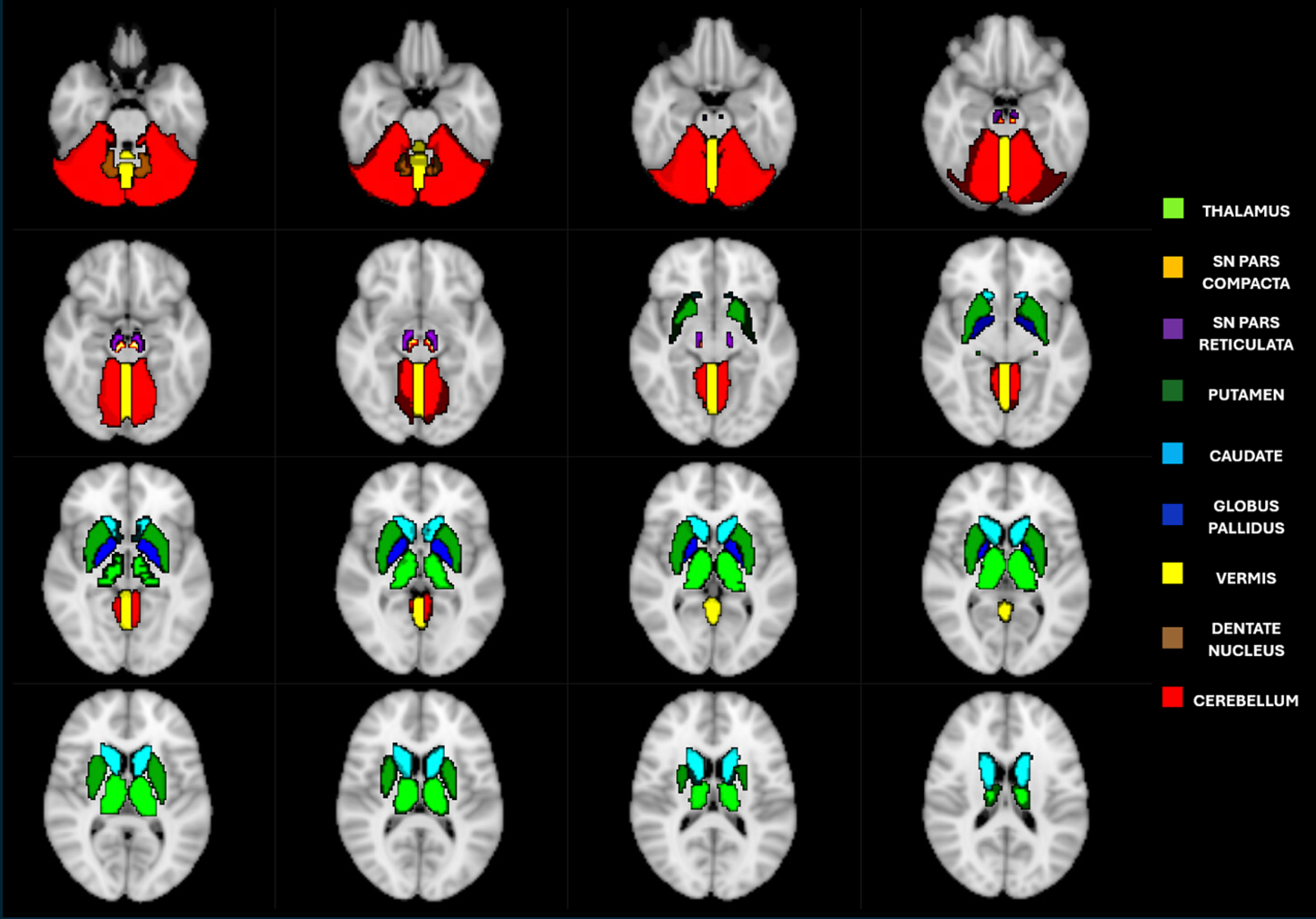


**Supplementary figure 2**. Masks for the left (L) and right (R) substantia nigra pars compacta and reticulata, thalamus, caudate, putamen, globus pallidum, cerebellum and vermis from the AAL3 atlas (Rolls et al., 2020), masks for the left and right dentate nucleus from the cerebellar probabilistic atlas (Diedrichsen et al., 2009) and mask for the whole cortex (Ct) from the probabilistic Harvard-Oxford cortical FSL atlas (https://www.fmrib.ox.ac.uk/fsl) in the standard MNI space.

**Supplementary figure 3**


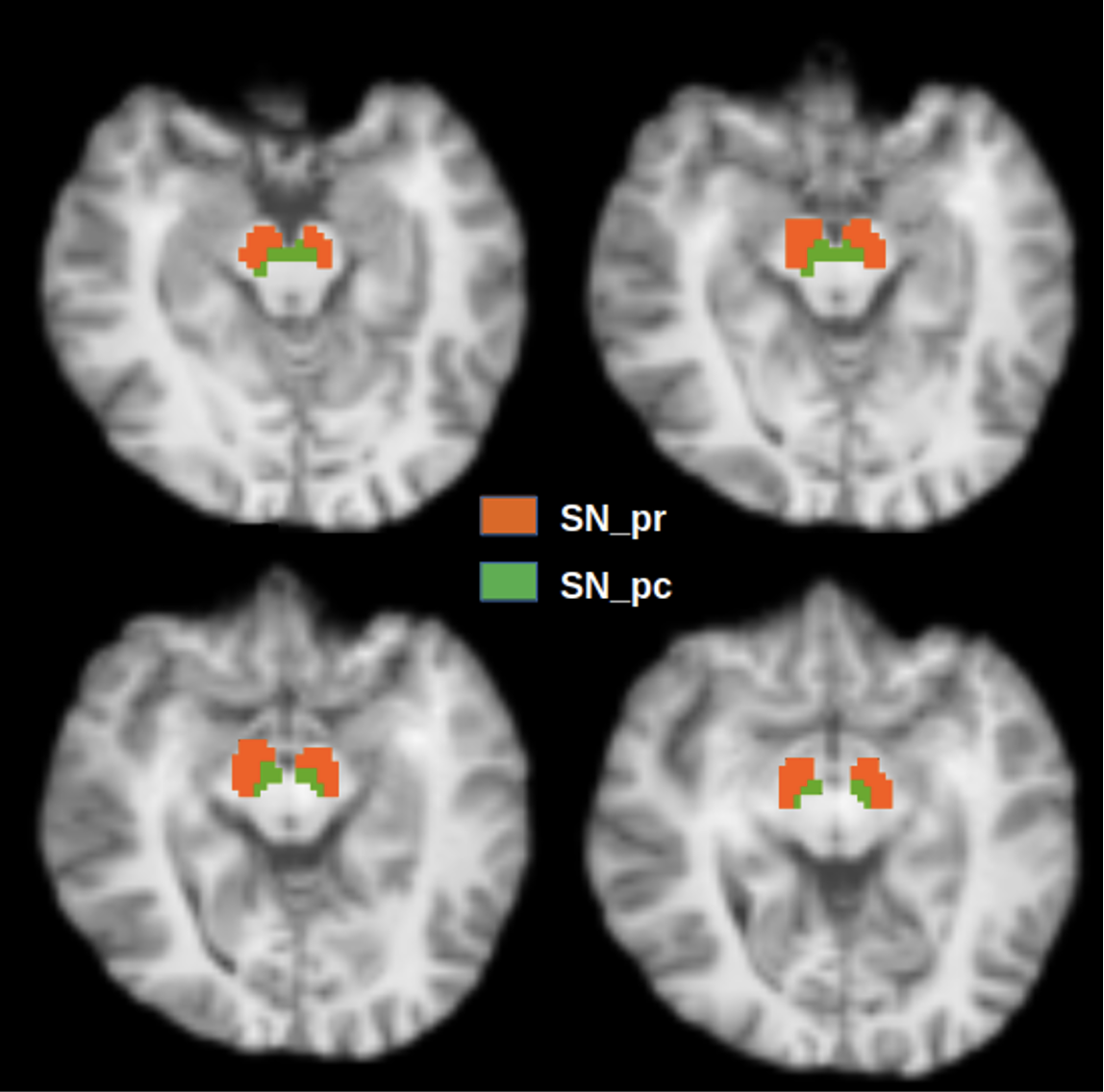


**Supplementary figure 3**. Individual masks of substantia nigra pars compacta (SN_pc) and pars reticulata (SN_pr) in the subject native space (for a representative subject).

**Supplementary figure 4**


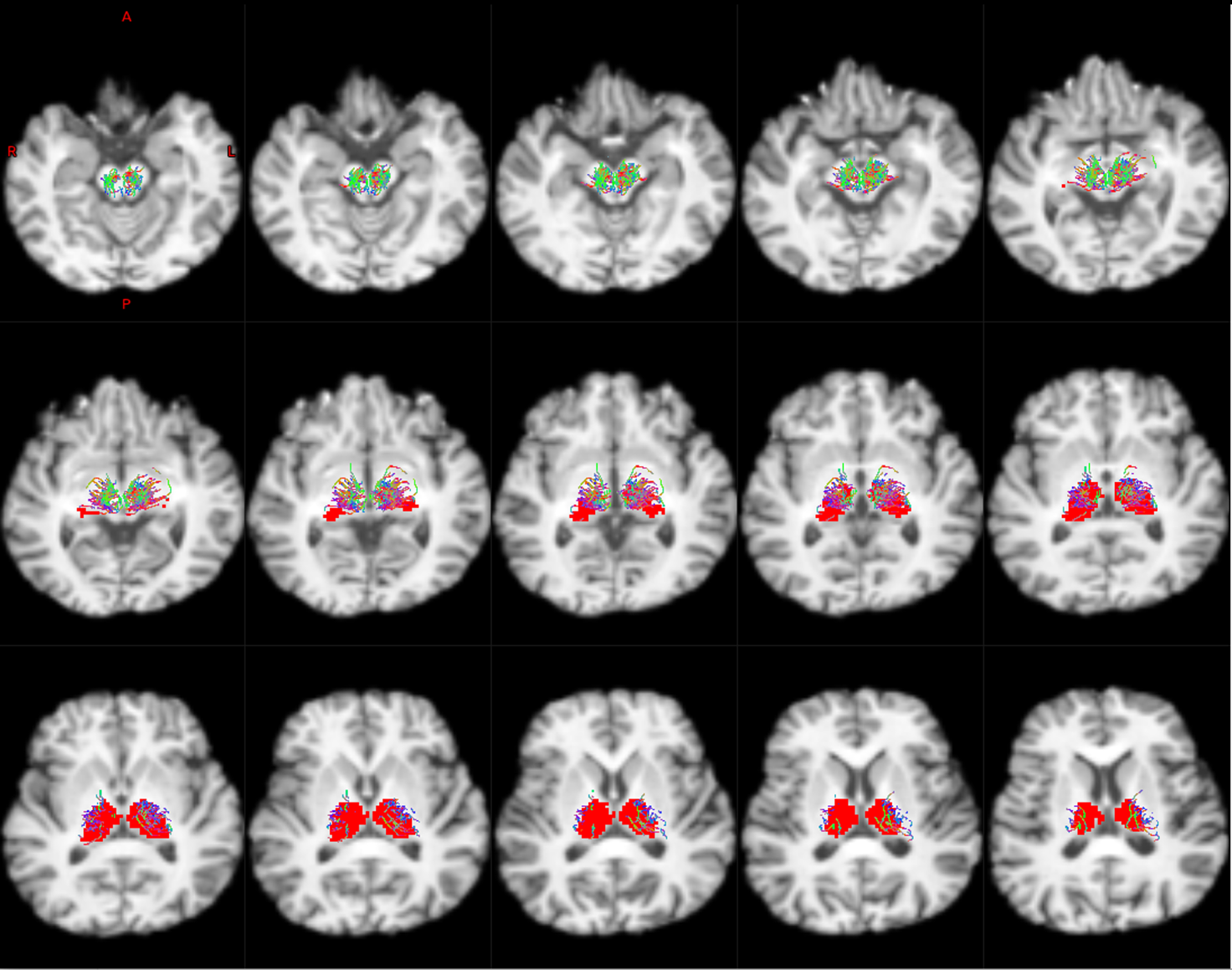


**Supplementary figure 4**. Selected streamlines directly connecting SNc (green mask) and thalamus (red mask) overlaid on axial slices from the 3D T1 scan for a representative subject as obtained from the whole-brain probabilistic tractography of MS1 data sets.

**Supplementary figure 5**.


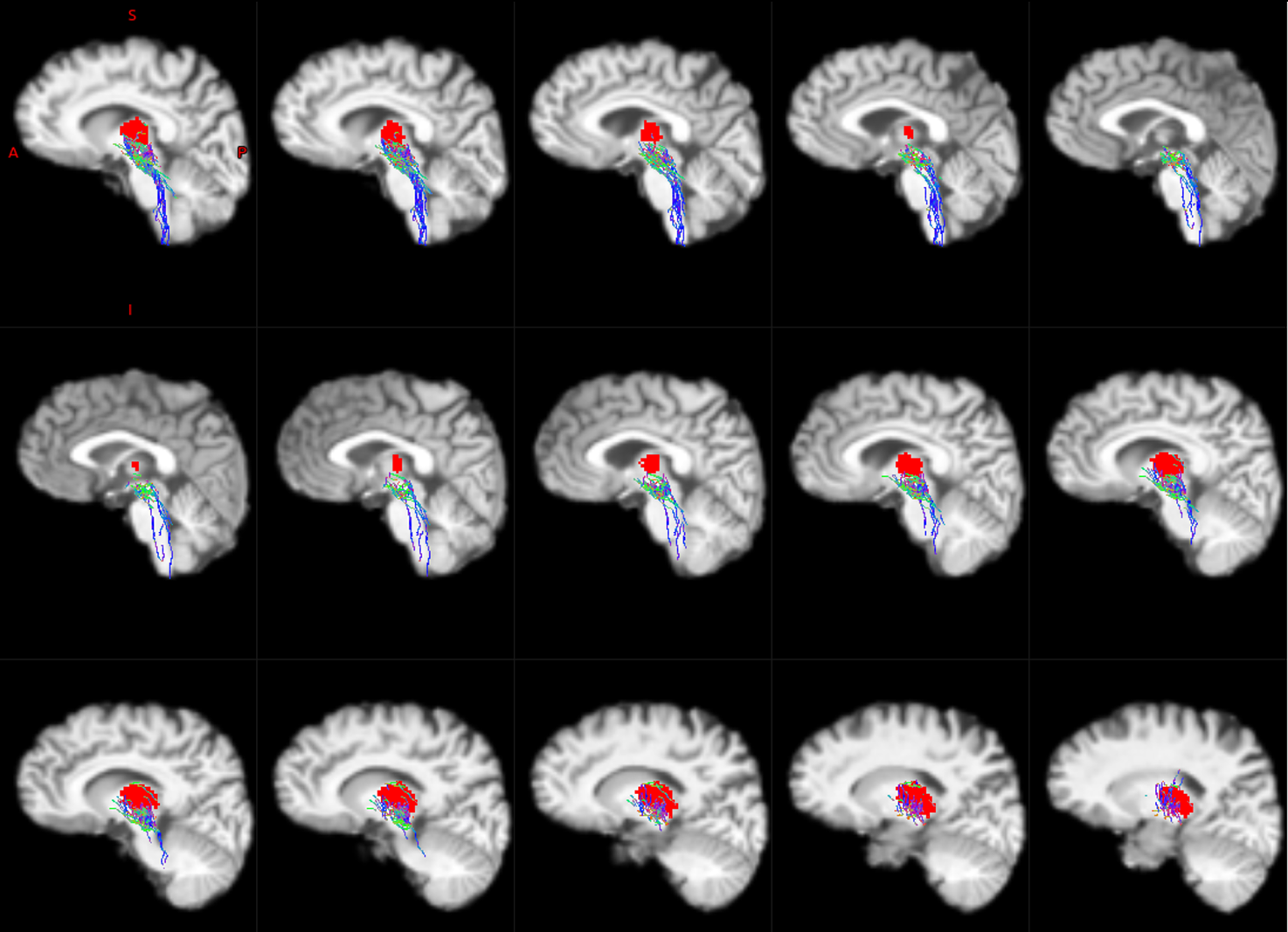


**Supplementary figure 5**. Selected streamlines directly connecting SNc (green mask) and thalamus (red mask) overlaid on sagittal slices from the 3D T1 scan for a representative subject as obtained from the whole-brain probabilistic tractography of MS1 data sets.

**Supplementary figure 6.**


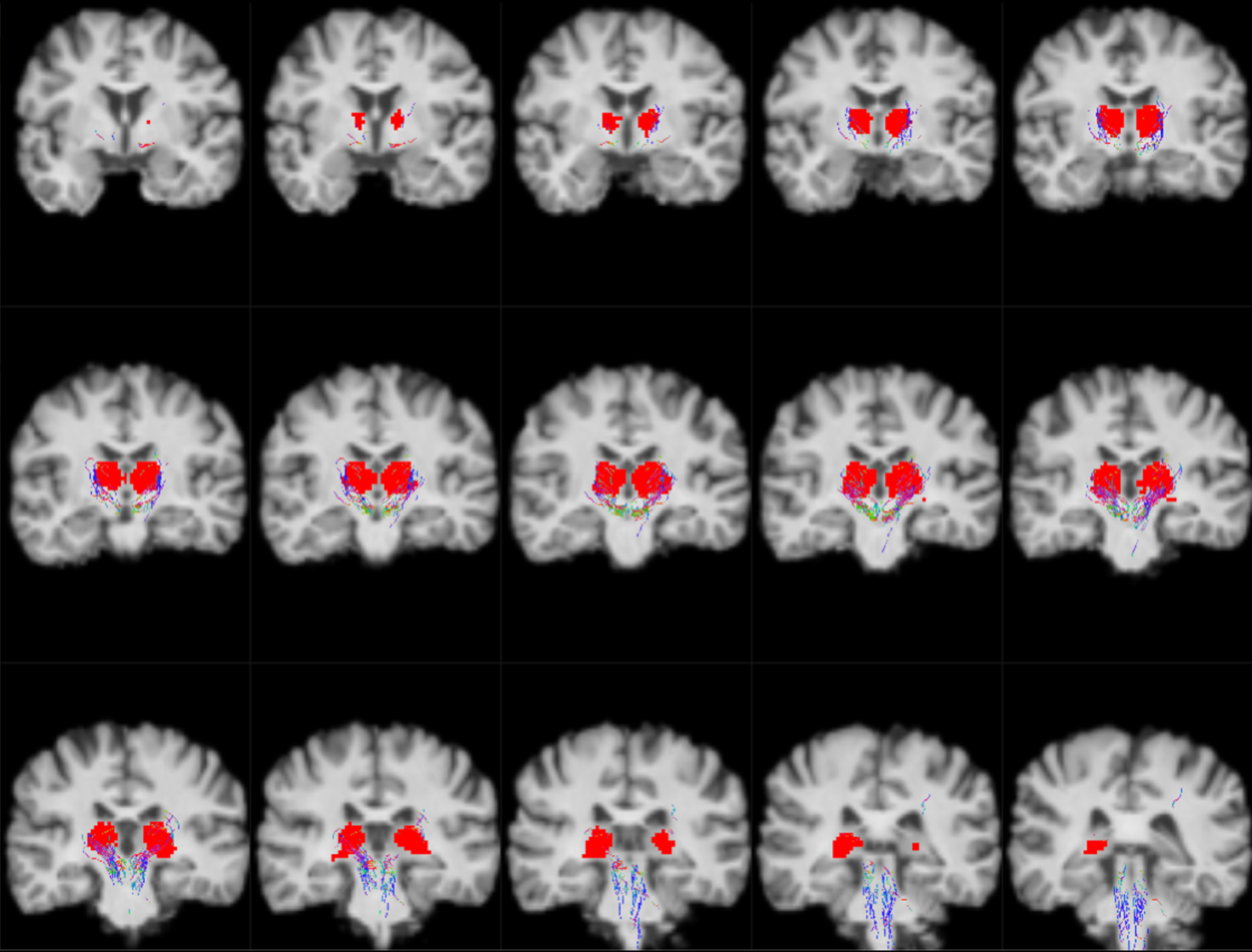


**Supplementary figure 6**. Selected streamlines directly connecting SNc (green mask) and thalamus (red mask) overlaid on coronal slices from the 3D T1 scan for a representative subject as obtained from the whole-brain probabilistic tractography of MS1 data sets.

**Supplementary figure 7**


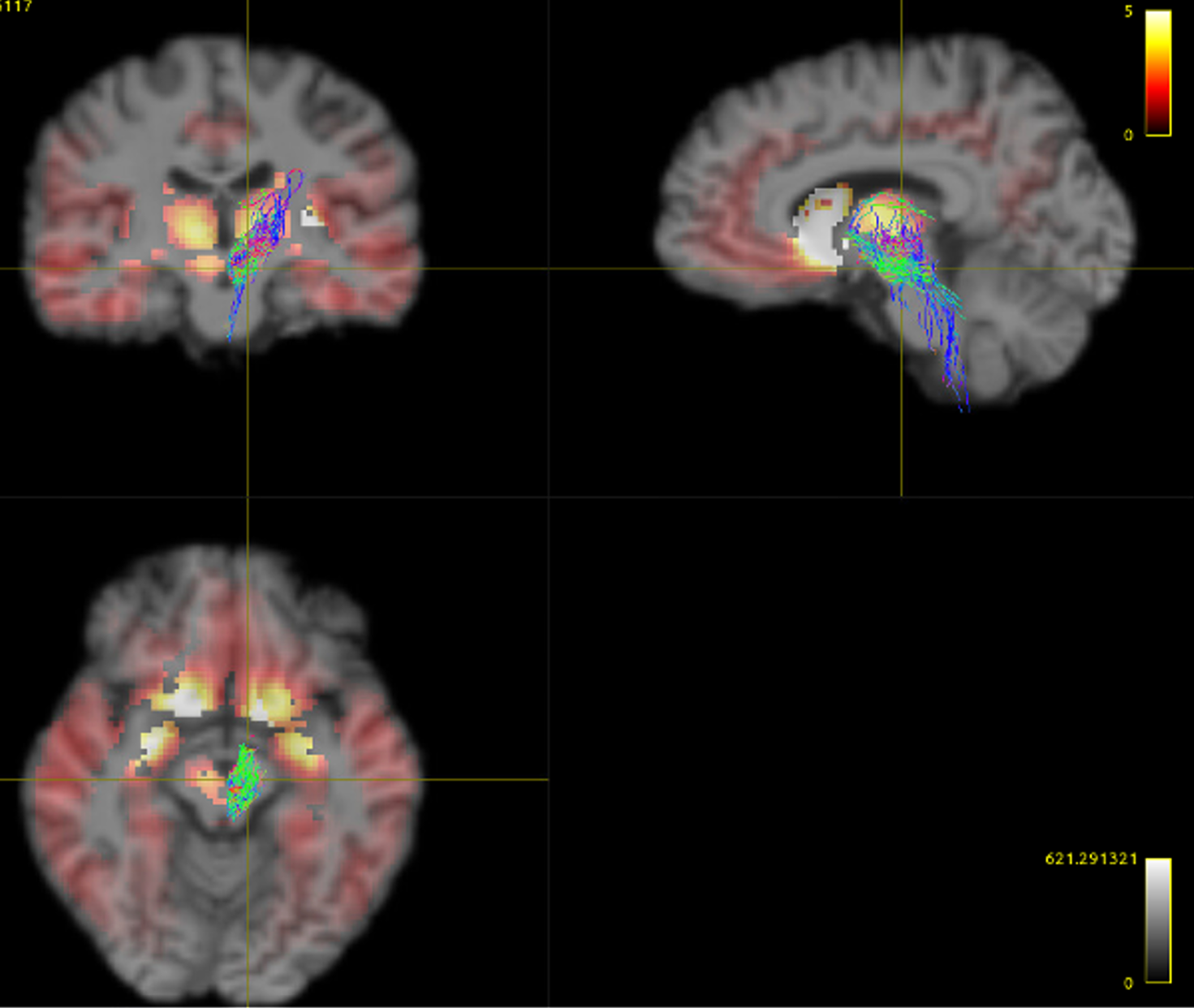


**Supplementary figure 7**. Selected streamlines directly connecting left SNc and left thalamus overlaid on a tri-planar view from the 3D T1 scan of one representative subject as obtained from the whole-brain probabilistic tractography of the MS1 data set. The first principal component map of dopamine receptor density from a normative PET data set back-transformed to the same 3D T1 scan is overlaid in transparency after scaling between z=0 and z=5.
